# Optimization of synergism of a recombinant auxiliary activity 9 from *Chaetomium globosum* with cellulase in cellulose hydrolysis

**DOI:** 10.1007/s00253-015-6592-3

**Published:** 2015-05-05

**Authors:** In Jung Kim, Ki Hyun Nam, Eun Ju Yun, Sooah Kim, Hak Jin Youn, Hee Jin Lee, In-Geol Choi, Kyoung Heon Kim

**Affiliations:** Department of Biotechnology, Korea University Graduate School, Seoul, 136-713 Republic of Korea; Pohang Accelerator Laboratory, Pohang University of Science and Technology, Pohang, 790-784 Republic of Korea

**Keywords:** AA9, GH61, Synergism, Cellulose hydrolysis, Cellulase, *Chaetomium globosum*

## Abstract

Auxiliary activity family 9 (AA9, formerly known as glycoside hydrolase family 61 or polysaccharide monooxygenase) is a group of fungal proteins that were recently found to have a significant synergism with cellulase in cellulose hydrolysis via the oxidative cleavage of glycosidic bonds of cellulose chains. In this study, we report the active expression of a recombinant fungal AA9 from *Chaetomium globosum* (*Cg*AA9) in a bacterial host, *Escherichia coli*, and the optimization of its synergistic activity in cellulose hydrolysis by using cellulase. The recombinant *Cg*AA9 (0.9 mg/g cellulose) exhibited 1.7-fold synergism in the hydrolysis of Avicel when incubated with 0.9 filter paper units of Celluclast 1.5 L/g cellulose. The first study of the active expression of AA9 using a bacterial host and its synergistic optimization could be useful for the industrial application of AA9 for the saccharification of lignocellulose.

**Electronic supplementary material:**

The online version of this article (doi:10.1007/s00253-015-6592-3) contains supplementary material, which is available to authorized users.

## Introduction

Lignocellulose is a promising feedstock for the production of biofuels or biochemicals. Due to the inherent recalcitrance of lignocellulose, its enzymatic hydrolysis, a key step to obtain fermentable sugar, is considered as a bottleneck in biofuel production (Lynd et al. 2008). Many efforts have been made to develop an efficient hydrolysis method for cellulose, which include the exploitation of synergism among proteins in cellulose hydrolysis (Arantes and Saddler 2010). The role of non-hydrolytic proteins in cellulose hydrolysis was proposed in the C_1_–C_x_ model several decades ago, in which the C_1_ factor (non-hydrolytic protein) acts as an enhancing protein for the C_x_ factor (hydrolytic enzyme) (Din et al. 1994; Gilligan and Reese 1954; Reese et al. 1950). Synergism between non-hydrolytic proteins and hydrolytic enzymes has been observed with carbohydrate-binding modules (CBMs), bacterial expansin, swollenin, and auxiliary activity 9 (AA9) (Dimarogona et al. 2012; Din et al. 1991; Harris et al. 2010; Kim et al. 2009; Lin et al. 2013; Suwannarangsee et al. 2012; Zhou et al. 2011).

AA9, formerly known as glycoside hydrolase family 61 (GH61) or polysaccharide monooxygenase (PMO), is a recently discovered and newly classified enzyme family in the Carbohydrate-Active enZymes (CAZy) database (Levasseur et al. 2013; Lombard et al. 2014). The AA9 proteins are widespread in cellulolytic fungi and have been shown to play a crucial role in cellulose degradation (Harris et al. 2010; Vaaje-Kolstad et al. 2010). It has been found that fungal AA9 and AA10 (CBM33) possess synergistic activity with cellulase and act by chemically modifying cellulose (Forsberg et al. 2011; Langston et al. 2011; Phillips et al. 2011a; Quinlan et al. 2011; Vaaje-Kolstad et al. 2010).

The cleavage action of the AA9 on cellulose requires a metal cofactor for activity and takes place via an oxidoreductive mechanism, yielding oxidized oligosaccharides at their reducing (aldonic acids) or non-reducing ends (4-ketoaldose in the case of C_4_ oxidation) (Beeson et al. 2012; Forsberg et al. 2011; Langston et al. 2011; Phillips et al. 2011a; Quinlan et al. 2011; Vaaje-Kolstad et al. 2010; Westereng et al. 2011). Due to their oxidoreductive action, cleavage reactions by AA9s are performed in the presence of electron donors such as ascorbic acid, reduced glutathione, or gallate (Beeson et al. 2012; Forsberg et al. 2011; Langston et al. 2011; Phillips et al. 2011a; Quinlan et al. 2011; Vaaje-Kolstad et al. 2010; Westereng et al. 2011). This oxidoreductive action on cellulose is exemplified by AA9s from *Thermoascus aurantiacus* (*Ta*GH61A) (Quinlan et al. 2011), *Phanerochaete chrysosporium* (*Pc*GH61D) (Westereng et al. 2011), and *Neurospora crassa* (*Nc*PMO-1 and *Nc*PMO-2) (Beeson et al. 2012; Phillips et al. 2011b). AA9 proteins are hypothesized to recognize the crystalline surface of cellulose for cleavage ahead of the action of classical hydrolases, thus expressing synergism with cellulase (Quinlan et al. 2011). The first report on the synergistic activity of AA9 with cellulase was shown with *Ta*GH61A and *Tt*GH61E (Harris et al. 2010); these proteins exhibited 1.3-fold and 1.1-fold synergistic activities with *Trichoderma reesei* cellulases on lignocellulose at ~70 % of the cellulose hydrolysis level. However, the synergistic activities of *Ta*GH61A and *Tt*GH61E were not observed on pure cellulose at cellulose hydrolysis levels over ~70 % glucose yield (Harris et al. 2010). Furthermore, in other studies, various AA9s such as *St*Cel61A from *Sporotrichum thermophile* (Dimarogona et al. 2012), *Pc*GH61D from *P. chrysosporium* (Westereng et al. 2011), and AA10 from *Streptomyces coelicolor* A3 (2) (Forsberg et al. 2011) showed synergism with cellulase in the hydrolysis of lignocellulose or pure cellulose. The synergistic activities of AA9s significantly differ depending on the type of substrate (lignocellulose vs. pure cellulose) and the genetic source of AA9.

To date, among the numerous AA9s that are widespread in fungi, only a few AA9s have been functionally characterized (Dimarogona et al. 2012; Forsberg et al. 2011; Harris et al. 2010; Westereng et al. 2011). Moreover, only AA10, which originates from a bacterium, has been actively expressed in a bacterial host (Forsberg et al. 2011; Vaaje-Kolstad et al. 2010). Other AA9s of fungal origin have not been reported to date as being actively expressed in bacterial hosts (Bey et al. 2013; Dimarogona et al. 2012; Harris et al. 2010; Phillips et al. 2011a; Westereng et al. 2011). In addition, no study has been published on the systematic optimization of reaction conditions for AA9 synergism. Efforts are needed towards the identification and functional study of additional AA9s that can be actively expressed in bacterial hosts and optimization of their synergistic activities.

*Chaetomium globosum*, a soil fungus belonging to ascomycetes, recently received much attention due to its high cellulolytic activity (Lakshmikant and Mathur 1990; Longoni et al. 2012). In particular, the AA9-encoding genes of *C. globosum* were found to be highly expressed when grown on cellulosic substrates (Longoni et al. 2012). Here, we report the first AA9 from *C. globosum* (*Cg*AA9) that is functionally active as a synergistic protein for cellulase. We have investigated the synergistic activity of recombinant *Cg*AA9, produced in *Escherichia coli*, on cellulose. The active expression of fungal AA9 in a bacterial host and its synergistic optimization in our study will pave the way for further studies on AA9s and their industrial applications.

## Materials and methods

### Reverse transcription polymerase chain reaction

The fungal strain *C. globosum* CBS 148.51 was obtained from the Rural Development Administration, Suwon, Korea. A single colony of *C. globosum* was cut into a circle with a 1-cm diameter, and the cut circle was inoculated into 20 mL malt extract broth. After cultivation for 19 h, 1 mL seed culture was inoculated into 100 mL medium containing 0.5 % (*w*/*v*) carbon source (glucose or Avicel), 0.2 % (*w*/*v*) NaNO_3_, 0.1 % (*w*/*v*) KH_2_PO_4_, 0.05 % (*w*/*v*) MgSO_4_ · 7H_2_O, 0.05 % (*w*/*v*) KCl, and 0.001 % (*w*/*v*) FeSO_4_ · 7H_2_O. Fungal hyphae were harvested from 100 mL culture by filtering through a filtration cloth (22–25 μm; Calbiochem, La Jolla, CA) and then washed with distilled water to remove residual medium components. The filtered hyphae were ground by a mortar and pestle under liquid nitrogen. The ground hyphae were disrupted by sonication to extract total RNA using an RNA isolation kit (Sigma-Aldrich, St. Louis, MO). The integrity of the extracted RNA was confirmed on agarose gel electrophoresis, and a complementary DNA (cDNA) library was constructed from 1 μg of total RNA using Maxime RT PreMix containing oligo dT primers (Intron Biotechnology, Seoul, Korea) following the manufacturer’s instructions. A cDNA library was constructed by incubating the reaction mixture at 45 °C for 60 min, followed by the inactivation of reverse transcriptase at 95 °C for 5 min. The genes for *Cg*AA9 (CHGG_09805; GenBank accession no. EAQ83401) and histone H4.2 (GenBank accession no. EAQ83894) as the control were amplified by the polymerase chain reaction (PCR) using gene-specific primers. The sequences of the forward and backward primers for *Cg*AA9 and histone H4.2 were 5′-GAAGGAGATATAAGGATGCACTACACCTTCCCGAGGCTA-3′ and 5′-ATGATGGTG ATGGTGAGCCGCTCCACACGGCCGGTC-3′, and 5′-ACTGGACGCGGAAAGGGCGG-3′ and 5′-GCCGCCGAAGCCGTAGAGAGT-3′, respectively. PCR was performed using SolgTM 2× Taq PCR smart mix 2 (SolGent, Daejeon, Korea) by the following program: 1 cycle at 95 °C for 2 min, 35 cycles including three steps for each cycle (95 °C for 20 s, 51 °C for 40 s, and 72 °C for 1 min), and one final cycle at 72 °C for 5 min. The transcript level was qualitatively visualized on a 0.8 % (*w*/*v*) agarose gel.

### Sequence alignment and structural modeling

The sequence alignment of *Cg*AA9 (GenBank accession no. EAQ83401), *Ta*GH61A (GenBank no. ACS05720) from *T. aurantiacus*, *Tt*GH61E (GenBank no. ACE10234) from *Thielavia terrestris*, *Hj*GH61B (GenBank no. AY281372) from *Hypocrea jecorina*, and *Nc*PMO-2 (GenBank no. EAA32426) and *Nc*PMO-3 (GenBank no. EAA33178) from *N. crassa*, was generated using Clustal W (Larkin et al. 2007) and ESPript (Gouet et al. 1999). The homology model of the *Cg*AA9 was constructed from the crystal structure of *Tt*GH61E (PDB code: 3EII). SWISS-MODEL was used as the protein structure modeling server (http://swissmodel.expasy.org/). All atom-contacts and geometry were checked using MolProbity (Chen et al. 2010), and protein structures were illustrated using PyMOL (http://www.pymol.org).

### Expression and purification of *Cg*AA9

The putative signal peptide sequence (Met1-Ala17) of *Cg*AA9 was analyzed by neural networks and hidden Markov models (Bendtsen et al. 2004), trained on eukaryotes using the SignalP 3.0 server (http://www.cbs.dtu.dk/services/SignalP-3.0/), and excluded from the whole sequence in this study. A codon-optimized CHGG_09805 gene (GenBank accession no. KJ668599) for expression in *E. coli* was synthesized (Bioneer, Daejeon, Korea), and the synthesized gene was cloned into the pET21a expression vector (Novagen, Darmstadt, Germany). The recombinant gene was transformed into competent *E. coli* BL21 (DE3) cells (Thermo Fisher Scientific, Waltham, MA), the protein expression of CHGG_09805 was induced by adding 0.5 mM isopropyl β-d-1-thiogalactopyranoside, and the culture was then maintained at 16 °C for 16 h. Recombinant *Cg*AA9 was purified from the endogenous proteins of the *E. coli* cells by affinity chromatography using a His-Trap column (GE Healthcare, Little Chalfont, UK). Briefly, cell pellets obtained by centrifugation at 2000*g* for 20 min were resuspended in equilibrium buffer (20 mM sodium phosphate and 500 mM NaCl; pH 7.4) and disrupted by sonication. The sonicated cell suspension was centrifuged at 30,000*g* for 30 min, and the supernatant containing soluble crude enzyme was loaded onto the His-Trap column, which was pre-equilibrated with the equilibrium buffer. After stepwise washing steps using different concentrations of imidazole buffers (20 mM sodium phosphate and 500 mM NaCl containing 0, 10, 25, 50, or 75 mM imidazole; pH 7.4), the proteins were eluted using elution buffer (20 mM sodium phosphate, 500 mM NaCl, and 100 mM imidazole; pH 7.4). The expression and purity of recombinant *Cg*AA9 were visually analyzed by sodium dodecyl sulfate polyacrylamide gel electrophoresis. The recombinant protein yield from the cell culture was approximately 1.5 mg/L.

### DPPH (2,2-diphenyl-1-picrylhydrazyl) assay

Reducing power was measured by means of DPPH assay. After mixing of 100 μL of DPPH radical at 0.2 mM (Sigma-Aldrich, St. Louis, MO) with 100 μL of a sample, the mixture was incubated for 30 min. The reducing power of the sample was determined by absorbance at 517 nm. Methanol was used as a blank, and 1 mM of antioxidants such as ascorbic acid, gallate, and reduced glutathione were used as positive controls.

### Synergistic hydrolysis by *Cg*AA9 with cellulase

To determine the synergistic activity of *Cg*AA9, 1 % (*w*/*v*) of Avicel PH 101 (Sigma-Aldrich, St. Louis, MO) was incubated with 1.8 filter paper units (FPU)/g Avicel of Celluclast 1.5 L (Novozymes, Bagsvaerd, Denmark) in the presence or absence of 1 mg of *Cg*AA9/g Avicel in 50 mM sodium acetate buffer (pH 5.0) in a total volume of 220 μL at 50 °C for 48 h. Sodium azide (NaN_3_) at 0.02 % (*w*/*v*) was added to all the reaction mixtures as an antibiotic. To terminate the enzyme reaction, the reaction mixture was heated at 95 °C for 5 min, followed by centrifugation at 30,000*g* for 5 min. The supernatant was used for sugar analysis with the 3,5-dinitrosalicylic acid (DNS) assay at 540 nm using d-glucose as the standard for reducing sugar. For the quantification of glucose and cellobiose, high-performance liquid chromatography (HPLC; Agilent 1100, Agilent Technologies, Waldbronn, Germany) was used with an HPX-87H column (Bio-Rad, Hercules, CA) at 65 °C, with 0.01 N H_2_SO_4_ at a flow rate of 0.5 mL/min as the mobile phase. Depending on the experiments, the amounts of Celluclast 1.5 L (0.045, 0.45, 0.9, and 3.6 FPU/g Avicel) and *Cg*AA9 (0, 0.9, 1.8, and 3.6 mg/g Avicel), temperature (20–70 °C), and pH (3.0–9.0) were varied. Synergism was determined as shown below: synergistic activity of *Cg*AA9 = reducing sugar yield produced by cellulase and *Cg*AA9/reducing sugar yield produced by cellulase alone.

## Results

### mRNA expression of *Cg*AA9 gene induced by cellulosic substrates

**Fig. 1:**
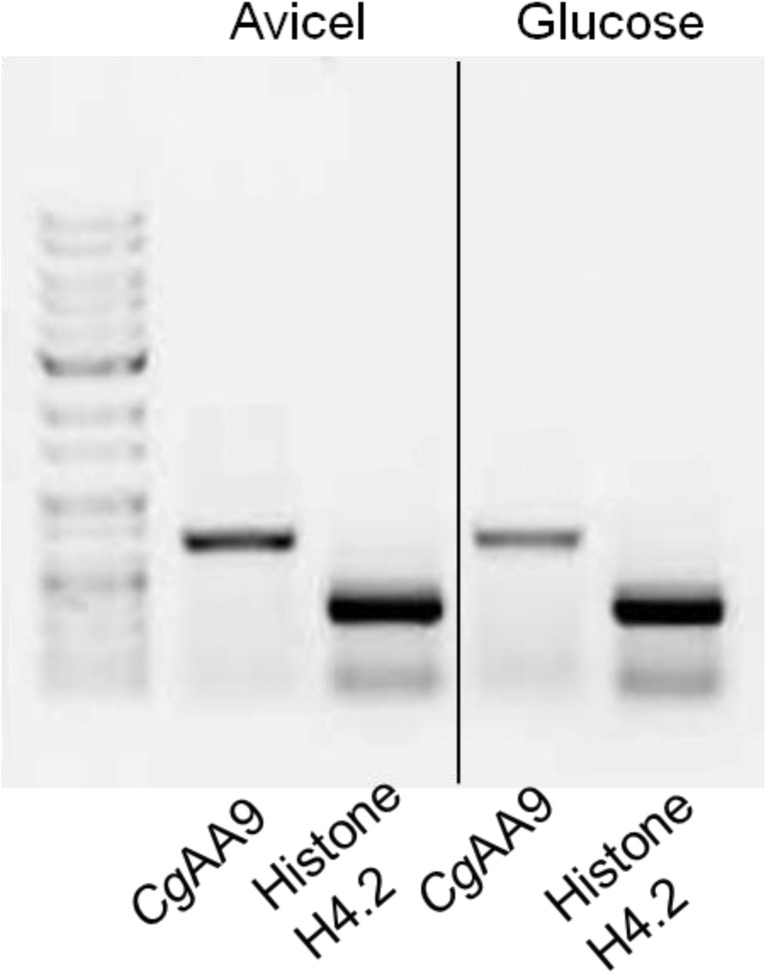
Upregulation of the CHGG_09805 gene induced by cellulosic substrates*. Lanes 1* and *2* indicate the amplified cDNA genes for CHGG_09805 and CHGG_10298 (histone H4.2), respectively, obtained from *C. globosum* grown on Avicel. *Lanes 3* and *4* indicate the amplified cDNA genes for CHGG_09805 and CHGG_10298 (histone H4.2), respectively, obtained from *C. globosum* grown on glucose

Induction of the expression of CHGG_09805, a candidate gene for AA9, by cellulosic substrates was examined by reverse transcription-polymerase chain reaction (RT-PCR). The cultivation profiles of *C. globosum* on different carbon sources (glucose, Avicel, and rice straw) showed higher growth rates on cellulosic substrates (i.e., Avicel and rice straw) than on glucose (Fig. S1 in the Supplementary Material). The band intensities for the amplified cDNA genes of CHGG_09805 from *C. globosum* grown on cellulose were higher than that for *C. globosum* grown on glucose when qualitatively visualized (Fig. 1). The band intensities for the amplified cDNA gene for histone H4.2 (CHGG_10298), which was used as the housekeeping gene, showed no significant differences regardless of whether *C. globosum* was grown on cellulose or glucose (Fig. 1). These results demonstrate that the messenger RNA (mRNA) expression of CHGG_09805 was strongly induced during growth on cellulosic substrates. This suggests that the protein encoded in CHGG_09805 is probably involved in cellulose degradation in *C. globosum*.

### Sequence alignment and homology modeling

**Fig. 2:**
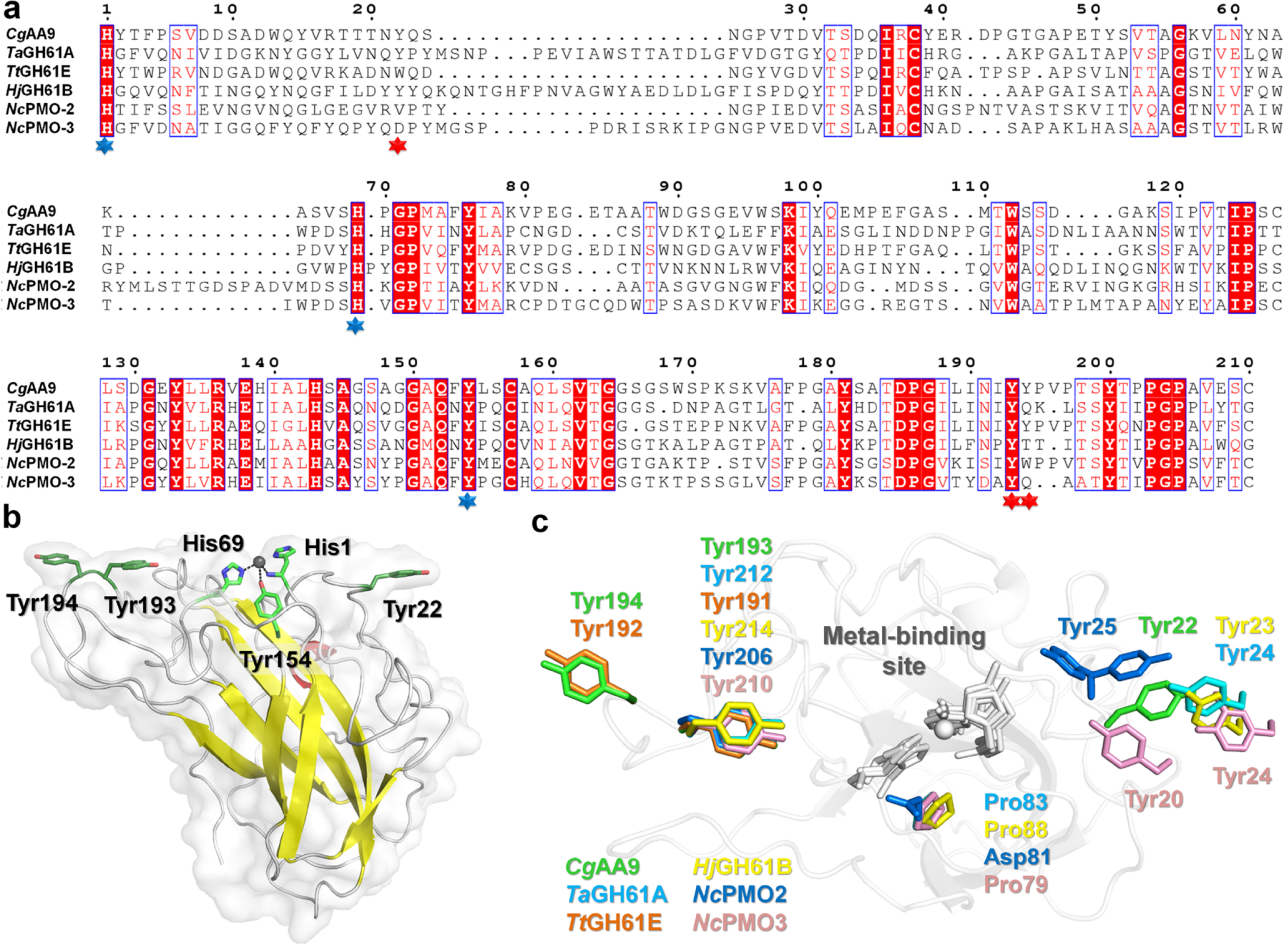
Sequence analysis and structural modeling of *Cg*AA9. **a** Sequence alignment of *Cg*AA9 (accession no.: EAQ83401) with other AA9s including *Ta*GH61A (ACS05720), *Tt*GH61E (ACE10234), *Hj*GH61B (AY281372), *Nc*PMO-2 (EAA32426), and *Nc*PMO-3 (EAA33178). The conserved residues are boxed in *red*, and the homologous residues are indicated by *unfilled boxes with red letters*. Residues involved in metal binding and putative substrate binding are marked with *blue and red asterisks*, respectively. The signal peptide sequence (Met1-Ala17) of *Cg*AA9 is not shown in the figure. **b** Cartoon representation of the structural model of *Cg*AA9. The functionally important residues are shown as sticks. The highly conserved metal-binding motifs (His1, His 69, and Tyr154) are shown as sticks in *green*. Putative substrate-binding sites (Tyr22, Tyr193, and Tyr194) are shown as sticks in *forest*. **c** The superimposition of putative substrate-binding site of AA9 homologs. *Cg*AA9, *Ta*GH61A (PDB code: 2YET), *Tt*GH61E (3EII), *Hj*GH61B (2VTC), *Nc*PMO2 (4EIR), and *Nc*PMO3 (4EIS) are indicated by *green*, *teal*, *orange*, *yellow*, *blue*, and *pink*, respectively. The metal-binding residues are indicated by *grey* sticks

To understand the potential function of *Cg*AA9 as the general activity of AA9, sequence alignment and homology modeling were performed. From the sequence alignment using other AA9s with known structures, the structure of *Cg*AA9 exhibited high similarity to those known AA9s compared here, especially to *Tt*GH61E (sequence identity of 61 %). In addition, amino acid residues that are reported to be highly conserved in other AA9s (His1, His69, and Tyr154) were also contained in *Cg*AA9 (Fig. 2a). These conserved residues are divalent metal-binding residues that are known to be important in enzyme activity (Harris et al. 2010; Karkehabadi et al. 2008; Li et al. 2012; Quinlan et al. 2011; Wu et al. 2013).

Homology modeling of *Cg*AA9 was performed using *Tt*GH61E as the template. The model structure of *Cg*AA9 was revealed to possess an immunoglobulin-like β-sandwich fold and the conserved metal-binding sites (His1, His69, and Tyr154) were observed on the surface. In addition, the putative substrate-binding or recognition sites (Tyr22, Tyr193, and Tyr194 in *Cg*AA9) that are linearly positioned with the metal-binding motif were observed (Fig. 2b). Next, the metal-binding motif and the putative substrate-binding residues of *Cg*AA9 were superimposed with those of other AA9 homologs (Fig. 2c). The three metal-binding sites and Tyr193 in *Cg*AA9 were structurally conserved among AA9 homologs, but Tyr194 in *Cg*AA9 was conserved only with Tyr192 in *Tt*GH61E. Meanwhile, Tyr22 in *Cg*AA9 was not observed with *Tt*GH61E, but this residue is considered functionally equivalent to Tyr24 in *Ta*GH61A, Tyr23 in *Hj*GH61B, Tyr25 in *Nc*PMO-2, or Tyr20 (or Tyr24) in *Nc*PMO-3 as the substrate-binding site.

### Synergism of *Cg*AA9 in cellulose hydrolysis by cellulase

**Fig. 3:**
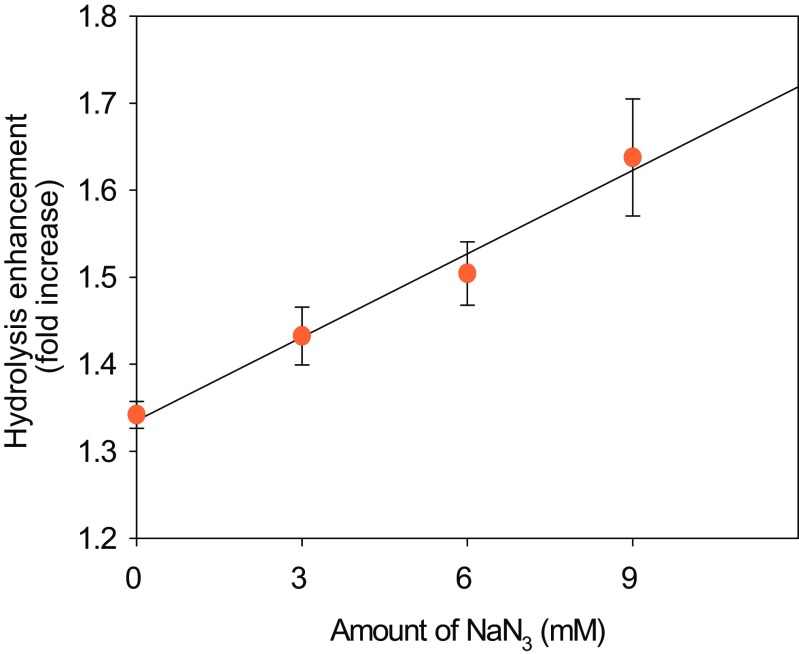
Effect of NaN_3_ on the synergistic activity of *Cg*AA9 with cellulase in the hydrolysis of cellulose. Avicel (1 %, *w*/*v*) was hydrolyzed by Celluclast 1.5 L (0.9 FPU/g Avicel) and/or *Cg*AA9 (1.8 mg/g Avicel) for 48 h at 50 °C in 50 mM sodium acetate buffer (pH 5.0). The amounts of NaN_3_ tested were 0, 3, 6, and 9 mM. Synergistic activity was represented as fold increase of reducing sugar generated from Avicel. Data are means ± standard errors

To determine the synergistic activity of recombinant *Cg*AA9 with cellulase, *Cg*AA9 was incubated with Celluclast 1.5 L and Avicel for cellulose hydrolysis. The synergism of *Cg*AA9 was measured without supplying any external electron donors throughout the experiments. This is because NaN_3_ (0.02 %, *w*/*v*) added as an antibiotic to the enzymatic reaction mixture was shown to act as a reducing cofactor for *Cg*AA9 (Figs. 3 and S2 in the Supplementary Material).

**Fig. 4:**
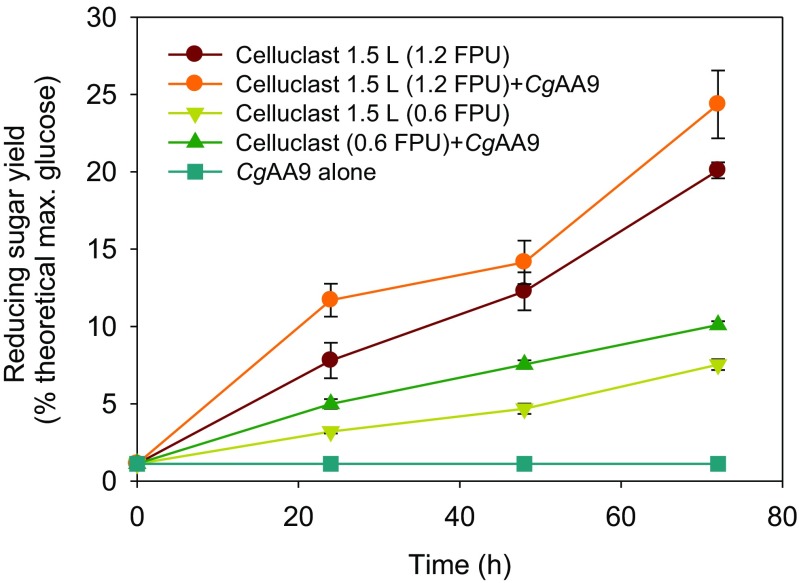
Time course of synergistic activity of *Cg*AA9 with cellulase in the hydrolysis of cellulose. Avicel (1 %, *w*/*v*) was hydrolyzed by Celluclast 1.5 L (0.6 and 1.2 FPU/g Avicel) and/or *Cg*AA9 (1.8 mg/g Avicel) in the presence of 0.02 % (*w*/*v*) of NaN_3_ for 0, 24, 48, and 72 h at 50 °C in 50 mM sodium acetate buffer (pH 5.0). Data are means ± standard errors

In the control experiment involving *Cg*AA9 alone with Avicel, *Cg*AA9 did not yield sugar products detectable by the DNS reagent. However, when *Cg*AA9 was added into the reaction mixture containing Celluclast 1.5 L and Avicel, the reducing sugar yield was significantly greater than that without *Cg*AA9 (Fig. 4). The synergism of *Cg*AA9 was demonstrated at two different loading concentrations (0.6 and 1.2 FPU/g cellulose) of Celluclast 1.5 L at all reaction times. To determine the feasibility of the actual application of accessory proteins, the cellulose hydrolysis level and cellulase loading level at which the synergism of the accessory protein is exhibited should be taken into account (Rudolf et al. 2008; Yang et al. 2006). In this study, synergism by *Cg*AA9 was observed when the cellulose hydrolysis yield was ~20 % of the theoretical maximum using 1.2 FPU of Celluclast 1.5 L/g Avicel. These levels of cellulose hydrolysis yield in this study are comparable to those commonly used for testing enzymatic digestibility or the simultaneous saccharification and fermentation (SSF) of lignocellulose (Rudolf et al. 2008; Yang et al. 2006). Since the major products of cellulose hydrolysis by Celluclast 1.5 L are glucose and cellobiose, the amounts of glucose and cellobiose from the hydrolysis of Avicel with Celluclast 1.5 L with or without *Cg*AA9 were quantified in this study (Fig. S3 in the Supplementary Material). The addition of *Cg*AA9 to the mixture of Celluclast 1.5 L and Avicel led to 1.3-fold and 1.2-fold increases in both glucose and cellobiose after 48 h, respectively (Fig. S3 in the Supplementary Material), compared to the control without *Cg*AA9.

### Effects of loading amounts of *Cg*AA9 and cellulase on synergism by *Cg*AA9

**Fig. 5:**
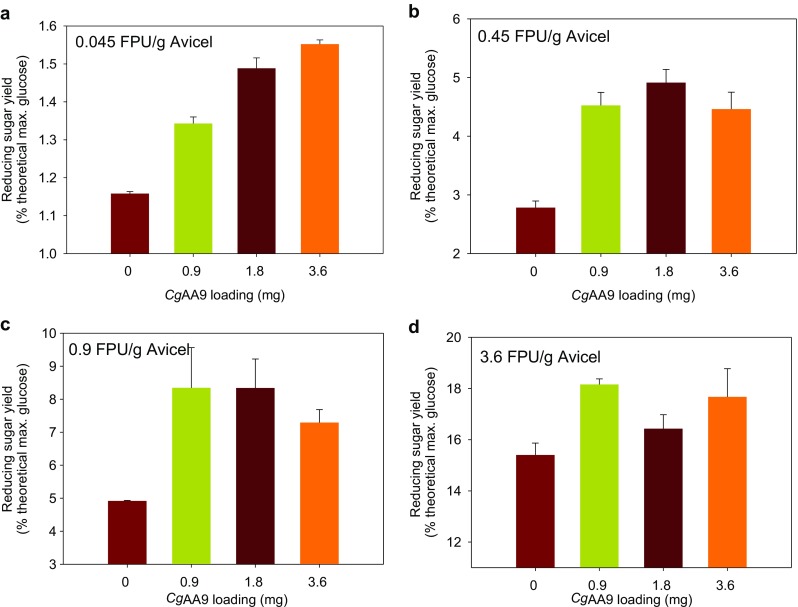
Effect of the loading of *Cg*AA9 and cellulase on their synergism in the hydrolysis of cellulose. Avicel (1 %, *w*/*v*) was hydrolyzed using four different concentrations of Celluclast 1.5 L: **a** 0.045, **b** 0.45, **c** 0.9, and **d** 3.6 FPU/g Avicel with or without four different concentrations of *Cg*AA9 at 0, 0.9, 1.8, and 3.6 mg/g Avicel in the presence of 0.02 % (*w*/*v*) of NaN_3_ for 48 h at 50 °C in 50 mM sodium acetate buffer (pH 5.0). Data are means ± standard errors

The mass ratio of an accessory protein and cellulase in their synergism is one of the key factors determining the economic feasibility of the accessory protein (Kim et al. 2009). The effects of the loading amounts of *Cg*AA9 and cellulase on synergism in cellulose hydrolysis were investigated in this study (Fig. 5). When *Cg*AA9 was added at 0.9 mg/g Avicel, significantly higher reducing sugar yields (1.3, 4.5, 8.3, and 18 % of the theoretical maximum of glucose, respectively; Fig. 5) were observed with all loadings of Celluclast 1.5 L (0.045, 0.45, 0.9, and 3.6 FPU/g Avicel) (*p* < 0.05) than in controls without *Cg*AA9. Overall, the highest extent of synergism (approximately 1.7-fold) was obtained with 0.45 or 0.9 FPU of Celluclast 1.5 L/g Avicel, regardless of the loading amount of *Cg*AA9 (Fig. 5b, c). When the Celluclast 1.5 L loading amount was 0.045 FPU/g Avicel, synergism increased with an increase in *Cg*AA9 loading (Fig. 5a). When the Celluclast 1.5 L loading was 0.45 or 0.9 FPU/g Avicel, the extent of synergism was not affected by varying the loading of *Cg*AA9 (*p* > 0.05) (Fig. 5b, c). On the contrary, when the cellulase loading was 3.6 FPU/g Avicel, at 1.8 and 3.6 mg/g Avicel, reducing sugar yields did not increase as much as at 0.9 mg/g Avicel. This is possibly because at higher cellulase (i.e., 3.6 FPU/g cellulose) and higher AA9 loadings (i.e., 1.8 and 3.6 mg/g cellulose), cellulase and *Cg*AA9 might have competed for cellulose as the substrate. Such phenomenon was also observed in other synergistic proteins (Kim et al. 2009).

### Effect of temperature and pH on synergism by *Cg*AA9

**Fig. 6:**
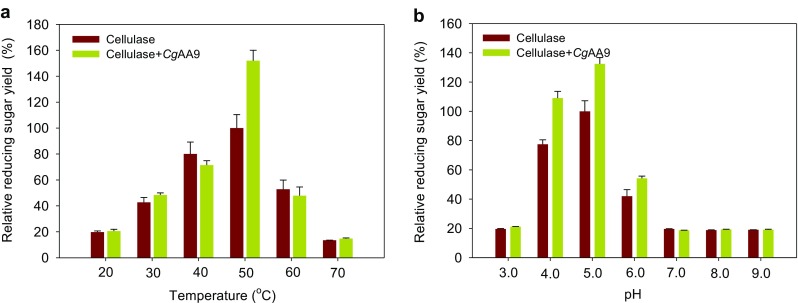
Effect of temperature and pH on the synergism of *Cg*AA9 and cellulase in the hydrolysis of cellulose. **a** Avicel (1 %, *w*/*v*) was hydrolyzed by 0.9 FPU of Celluclast 1.5 L/g Avicel with or without 1.8 mg of *Cg*AA9/g Avicel for 48 h at different temperatures of 20–70 °C in 50 mM sodium acetate buffer (pH 5.0). **b** Avicel (1 %, *w*/*v*) was hydrolyzed by 0.9 FPU of Celluclast 1.5 L/g Avicel with or without 1.8 mg of *Cg*AA9/g Avicel for 48 h at pH 3.0 to 8.0 at 50 °C; 50 mM sodium citrate buffer (for pH 3.0 to 6.0), 50 mM potassium phosphate buffer (for pH 7.0), 50 mM Tris-HCl buffer (for pH 8.0), and 50 mM glycine-NaOH for (pH 9.0) were used for different pH values. Experiments were conducted in the presence of 0.02 % (*w*/*v*) of NaN_3_. Data are means ± standard errors

To study the effect of temperature on the synergistic activity of *Cg*AA9, cellulose hydrolysis experiments were performed at various temperatures ranging from 20 to 70 °C (Fig. 6a). The production of reducing sugar from Avicel by Celluclast 1.5 L alone was strongly temperature-dependent, and the highest activity was exhibited at 50 °C, which is the optimal temperature of Celluclast 1.5 L. When *Cg*AA9 was incubated with cellulase, the synergistic activity of *Cg*AA9 was observed at 50 °C. At other temperatures, the synergistic activity of *Cg*AA9 was negligible or not observed. These results show that the synergism of *Cg*AA9 is highly dependent on the optimal temperature of cellulase. For industrial application, *Cg*AA9 is not appropriate for typical SSF, in which saccharification and fermentation processes are performed together at 38 °C. Instead, *Cg*AA9 could possibly be utilized in the hydrolysis step of the separate hydrolysis and fermentation (SHF) process because the typical temperature for hydrolysis is 50 °C.

To investigate the effect of pH on the synergism of *Cg*AA9 and cellulase, cellulose hydrolysis experiments were performed at different pH values ranging from 3.0 to 9.0 (Fig. 6b). Similar to the effect of temperature on the activity of cellulase alone, the activity of cellulase alone was highly dependent on pH; the maximal reducing sugar yield of Celluclast 1.5 L was seen at pH 5.0. When *Cg*AA9 was added to the hydrolysis reaction mixture, synergism was observed at pH 4.0 (1.4-fold), 5.0 (1.3-fold), and 6.0 (1.3-fold), whereas no significant synergism was seen at pH 3.0, 7.0, 8.0, and 9.0 (*p* > 0.05). The synergistic activity of *Cg*AA9 was exhibited only at the pH where the activity of Celluclast 1.5 L alone was adequately high. From these results, temperature and pH were found to be the key factors to be considered in the industrial utilization of *Cg*AA9 as synergistic protein.

### Synergistic or stabilizing effect of *Cg*AA9

**Fig. 7:**
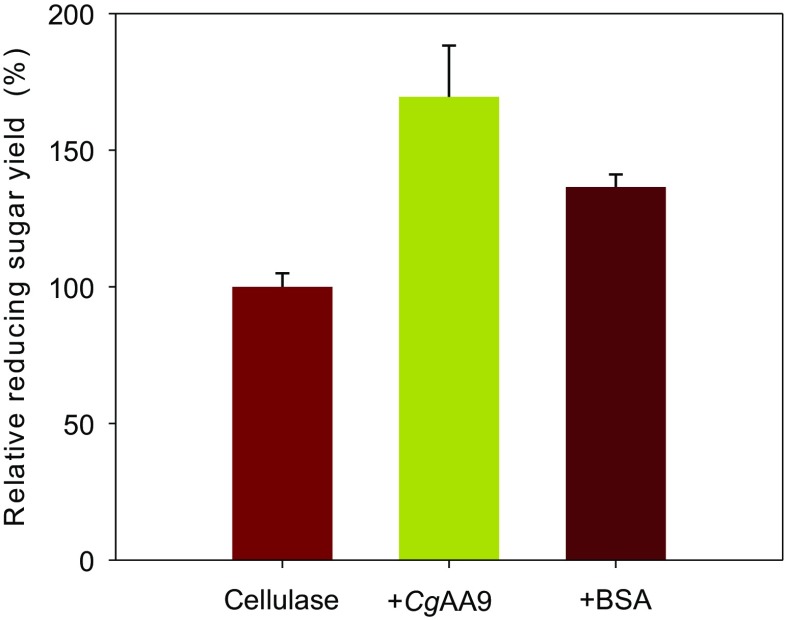
Comparison of the synergistic activities of *Cg*AA9 and BSA. Avicel (1 %, *w*/*v*) was hydrolyzed by 0.9 FPU of Celluclast 1.5 L/g Avicel with or without 1.8 mg of *Cg*AA9 or 4.8 mg of BSA/g Avicel in the presence of 0.02 % (*w*/*v*) of NaN_3_ for 48 h at 50 °C in 50 mM sodium acetate buffer (pH 5.0); equal molar masses of *Cg*AA9 and BSA were used. Data are means ± standard errors

To determine whether the increase in cellulose hydrolysis yield by *Cg*AA9 was due to a synergistic or stabilizing effect, bovine serum albumin (BSA) was selected as an additive control protein due to its extensive use in the stabilization of enzymes (Brethauer et al. 2011; Hu et al. 2011). In this study, comparing the effects of BSA and *Cg*AA9, the increase in cellulose hydrolysis yield by *Cg*AA9 was significantly greater (e.g., 1.7-fold) than that by the same molar mass of BSA (e.g., 1.3-fold) (*p* < 0.05) (Fig. 7).

## Discussion

AA9-encoding genes are distributed throughout a variety of cellulytic fungi, probably due to their essential role in cellulose degradation (Martinez et al. 2009; Phillips et al. 2011b; Tian et al. 2009; Vanden Wymelenberg et al. 2009). Especially, *C. globosum*, the soil fungus used as the genetic source of AA9 in this study, possesses more than forty-four AA9-encoding genes (Lakshmikant and Mathur 1990; Longoni et al. 2012), and CHGG_09805 (*Cg*AA9) is one of them. In this study, mRNA expression of CHGG_09805 was induced during growth on cellulosic compounds, implying its potential involvement in cellulose hydrolysis. Moreover, sequence analysis and structural homology modeling of CHGG_09805 revealed its potential synergistic activity as an AA9. Specifically, *Cg*AA9 shares conserved divalent metal-binding residues with other AA9s that are known to be important in its activity (His1, His69, and Tyr154) (Harris et al. 2010; Karkehabadi et al. 2008; Li et al. 2012; Quinlan et al. 2011; Wu et al. 2013). The homology modeling of *Cg*AA9 presented here suggests that these residues are plausible to exist on the surface of *Cg*AA9 and well superimposed on the corresponding residues of known structures of other AA9s (Harris et al. 2010; Karkehabadi et al. 2008; Li et al. 2012; Quinlan et al. 2011; Wu et al. 2013).

In this study, *Cg*AA9 expressed in *E. coli* showed synergistic activity in the hydrolysis of pure cellulose (Avicel) without adding an external reducing cofactor. This is because the reaction mixture for *Cg*AA9 possessed reducing power as revealed by the DPPH assay (Fig. S2 in the Supplementary Material). Of the components contained in the reaction mixture, NaN_3_ was found to be a major contributor to the overall reducing power of reaction mixture (Fig. 3). The extent of synergism by *Cg*AA9 increased with increasing the concentration of NaN_3_ from 0 to 9 mM. In several previous studies, AA9s showed no or little synergistic activity on pure cellulose without an external electron donor (Dimarogona et al. 2012; Cannella et al. 2012; Harris et al. 2010). However, when using pretreated lignocellulose, an external electron donor was not required since lignin in pretreated lignocellulose reducing power for AA9 (Dimarogona et al. 2012; Hu et al. 2014). Taken together, the requirement of an electron donor for AA9 was confirmed in this study. However, reaction products including native and oxidized sugars were not detected by the analysis of matrix-assisted laser desorption/ionization tandem time of flight/mass spectrometer (MALDI-TOF/TOF MS) when *Cg*AA9 alone without cellulase was incubated on cellulose (data not shown). No detection of reaction products by *Cg*AA9 was possibly due to the low enzymatic activity of *Cg*AA9.

In this study, a fungal AA9 was functionally expressed in a bacterial host. Previously, post-translational modifications including glycosylation and methylation were observed in some AA9s. Specifically, methylated His1 was observed in *Ta*GH61A (Quinlan et al. 2011), and PMOs from *N. crassa* (PMO-2 and PMO-3) were revealed to contain the sites of N-glycosylation (Li et al. 2012). However, the type and pattern of post-translational modifications in AA9s are random and diverse, and their functional significance on cellulose hydrolysis still remains elusive (Li et al. 2012). Main functions of AA9s such as synergistic activity and reducing cofactor-dependent activity were observed in *Cg*AA9 which was expressed in *E. coli* lacking post-translational modification. This result implies that bacterial expression of fungal AA9 is able to function even without post-translation modifications. Furthermore, a recent study reported that methylation of His1 had a minor effect on AA9’s activity (Kim et al. 2014). Still, the exact functional significance of methylation or glycosylation on AA9 requires further study. Although our fungal *Cg*AA9 expressed in *E. coli* functioned as an AA9, it cannot be ruled out that *Cg*AA9 could have shown higher synergism with post-translational modifications. It would be interesting to compare the activities of AA9s expressed in either prokaryotic or eukaryotic system in further study.

When determining the feasibility of the actual application of synergistic proteins, key determinants are extent of synergism, synergistic protein loading and hydrolysis yield (Rudolf et al. 2008; Yang et al. 2006). If the addition of a small amount of an accessory protein brings about a substantial increase in cellulase activity, the loading amount of cellulase can be reduced to lower the enzyme cost in the industrial process. In addition, the high yield of hydrolysis at which the synergism of the accessory protein is exhibited is required for the industrial conversion of cellulose. In this regard, synergistic performance of *Cg*AA9 was compared with those of other non-hydrolytic accessory proteins, which were selected based on their synergistic activity on pure cellulose (Table 1) (Dimarogona et al. 2012; Forsberg et al. 2011; Kim et al. 2009; Lee et al. 2010; Westereng et al. 2011). At a high hydrolysis yield when 0.9 mg of *Cg*AA9 was incubated with 3.6 FPU/g Avicel, the synergistic activity of *Cg*AA9 (1.2-fold) was comparable to that of *Pc*GH61D (1.3-fold) at a similar yield of cellulose hydrolysis (17–19 %). However, the protein loading of *Cg*AA9 required to achieve a similar extent of synergism was approximately 6.2 times lower than that for *Pc*GH61D in our study (Westereng et al. 2011).

**Table 1:** Comparison of the synergistic activity of *Cg*AA9 with Celluclast 1.5 L on crystalline cellulose with those of other synergistic proteins such as AA9s, AA10, and bacterial expansins

	Extent of synergism (fold)	Synergistic protein loading (mg/g cellulose)	Hydrolysis yield without synergistic protein (% theoretical max.)	Hydrolysis yield with synergistic protein (% theoretical max.)	Substrate
*Cg*AA9 (this study)	1.2	0.9	15.4	18.2	Avicel
	1.7	0.9	4.9	8.3	Avicel
*Pc*GH61D (Westereng et al. 2011)	1.3	5.6	13.6	17.3	Avicel
CBM33 (Forsberg et al. 2011)	2.5	4.0	3.6	9.1	Filter paper
*Bs*EXLX1 (Kim et al. 2009)	5.7	0.3	0.3	1.8	Filter paper
*Hc*EXLX2 (Lee et al. 2010)	5.1	1.3	0.7	3.6	Filter paper

For the comparison with synergistic proteins other than AA9, 0.9 mg of *Cg*AA9 with 0.9 FPU of Celluclast 1.5 L/g Avicel was chosen, at which the highest synergistic activity (1.7-fold) was shown in this study. When compared with that by CBM33, the synergism by a 4.4-fold lower loading amount of *Cg*AA9 was lower only by 1.5-fold than that of CBM33 to achieve a similar yield of cellulose hydrolysis (8–10 %) (Forsberg et al. 2011). When the synergism by *Cg*AA9 was compared to those of bacterial expansins such as *Bs*EXLX1 and *Hc*EXLX2, these two bacterial expansins exhibited synergism only at much lower loadings of Celluclast 1.5 L than did *Cg*AA9 in our study (Kim et al. 2009; Lee et al. 2010). Therefore, the synergistic activities of *Bs*EXLX1 and *Hc*EXLX2 are not industrially feasible because their synergism is seen at a low hydrolysis yield of cellulose. Overall, the synergism by *Cg*AA9 in this study is more practically applicable than those of other non-hydrolytic accessory proteins selected here. This is because the synergism is seen at lower loading levels of *Cg*AA9 or at a higher hydrolysis level of cellulose than in the case of other accessory proteins.

The reducing sugar yield enhanced by *Cg*AA9 was significantly higher than that by BSA. This implies that the increased reducing sugar by *Cg*AA9 is more than a stabilizing effect and is rather a synergistic effect. BSA is often added to enzyme reaction mixtures as a stabilizing agent due to its ability to reduce the deactivation of cellulases that occurs at the air-liquid interface (Brethauer et al. 2011; Hu et al. 2011; Kim et al. 1982). Likewise, the enhancing effect by BSA observed in this study is considered a stabilizing effect rather than a synergistic effect causing alterations in cellulose structure. Indeed, there is no report of structural disruption of cellulose by BSA (Brethauer et al. 2011; Hu et al. 2011). By contrast, the effect of *Cg*AA9 on cellulose hydrolysis in the present study may be due to the direct alteration of cellulose, probably caused by the PMO activity of *Cg*AA9. In conclusion, the first expression of a fungal AA9 in a bacterial host and the optimization for its synergism without the requirement for external electron donors will facilitate the development of synergistic proteins for the industrial application.

## Electronic supplementary material

ESM 1 (PDF 151 kb)
