# Supplementary material for: Optimization of synergism of a recombinant auxiliary activity 9 from Chaetomium globosum with cellulase in cellulose hydrolysis
Source: Appl Microbiol Biotechnol. 2015 May 5;99:8537–47. doi: 10.1007/s00253-015-6592-3 (PMC4768223; doi:10.1007/s00253-015-6592-3)
Supplement: Supplementary file 1 — (PDF 151 kb) [file 253_2015_6592_MOESM1_ESM.pdf]

Applied Microbiology and Biotechnology

Optimization of synergism of a recombinant auxiliary activity 9 from *Chaetomium globosum* with cellulase in cellulose hydrolysis

In Jung Kim[1], Ki Hyun Nam[2], Eun Ju Yun[1], Sooah Kim[1], Hak Jin Youn[1], Hee Jin Lee[1], In-Geol Choi[1], Kyoung Heon Kim[1]*

[1]Department of Biotechnology, Korea University Graduate School, Seoul 136-713, Republic of Korea
[2]Pohang Accelerator Laboratory, Pohang University of Science and Technology, Pohang 790-784, Republic of Korea

*Correspondence: khekim@korea.ac.kr

Supplementary Materials

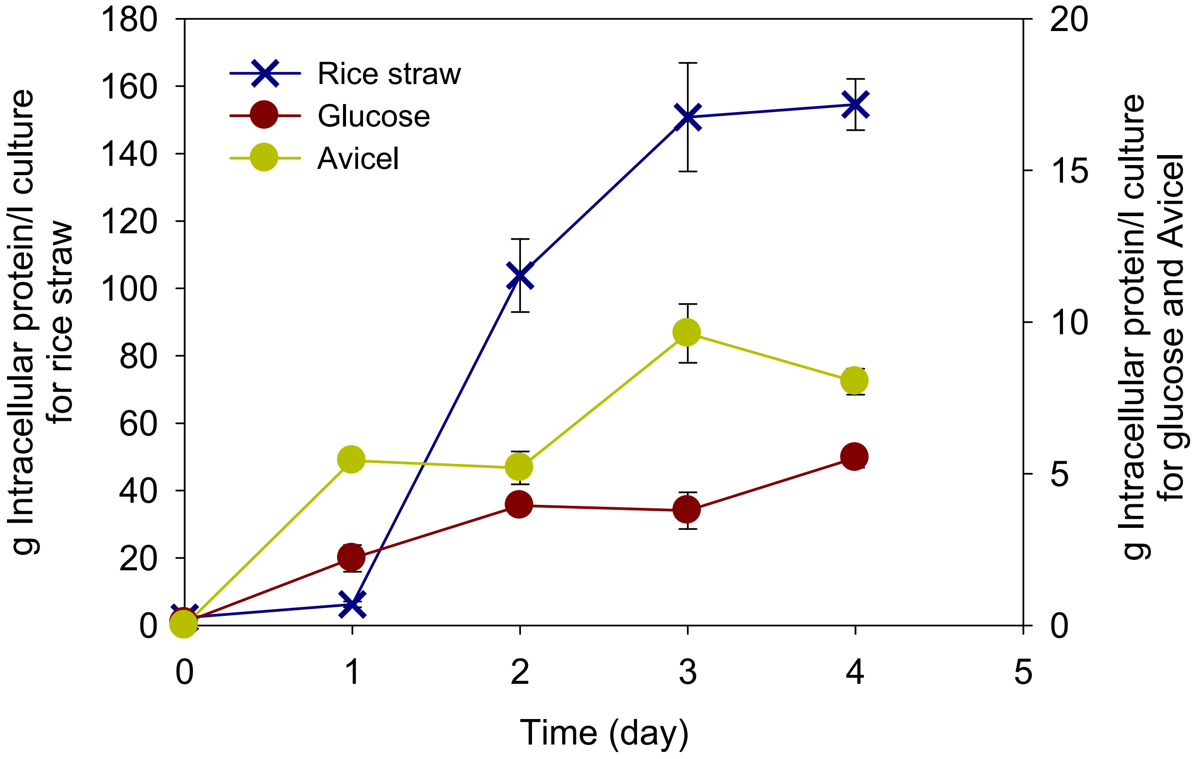

Fig. S1 Growth curve of *C. globosum* CBS 148.51 grown on glucose, Avicel, and rice straw. The magnitude of fungal growth is represented in terms of grams of intracellular protein per liter of culture from 1 to 4 day, which was extracted by disrupting cells using Branson Sonifier 450 (Branson Ultrasonics, Danbury, CT)

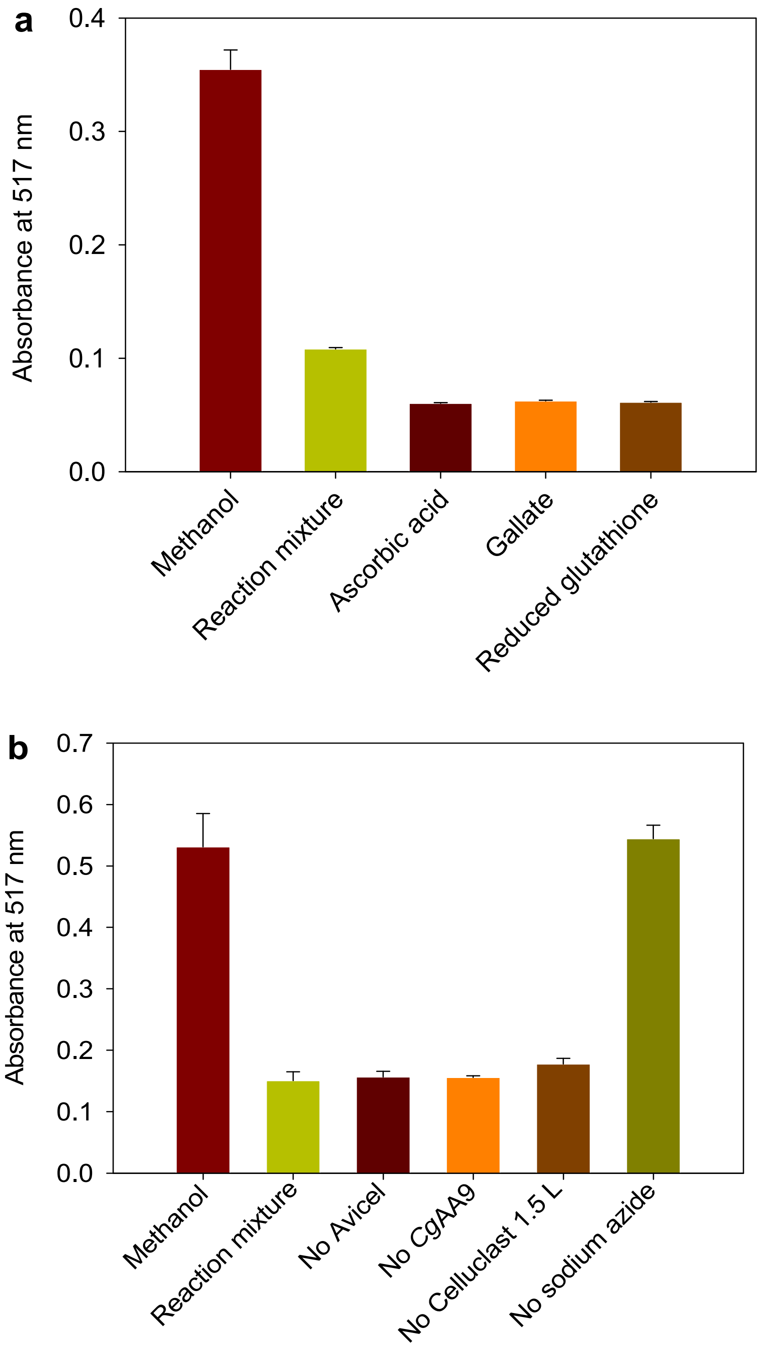

Fig. S2 Reducing power assay by means of DPPH method. **a** The reducing power of reaction mixture for *Cg*AA9 was measured by comparing with the absorbance at 517 nm of methanol as negative control, and those of ascorbic acid, gallate and reduced glutathione at 1 mM as positive controls. **b** The reducing powers of various reaction mixtures were compared to identify the major component in the reaction mixture for *Cg*AA9 which contributed to the

overall reducing power. Each component comprising the reaction mixture such as Avicel, *Cg*AA9, Celluclast 1.5 L or NaN_3 was subtracted from the reaction mixture. Data are means ± standard errors

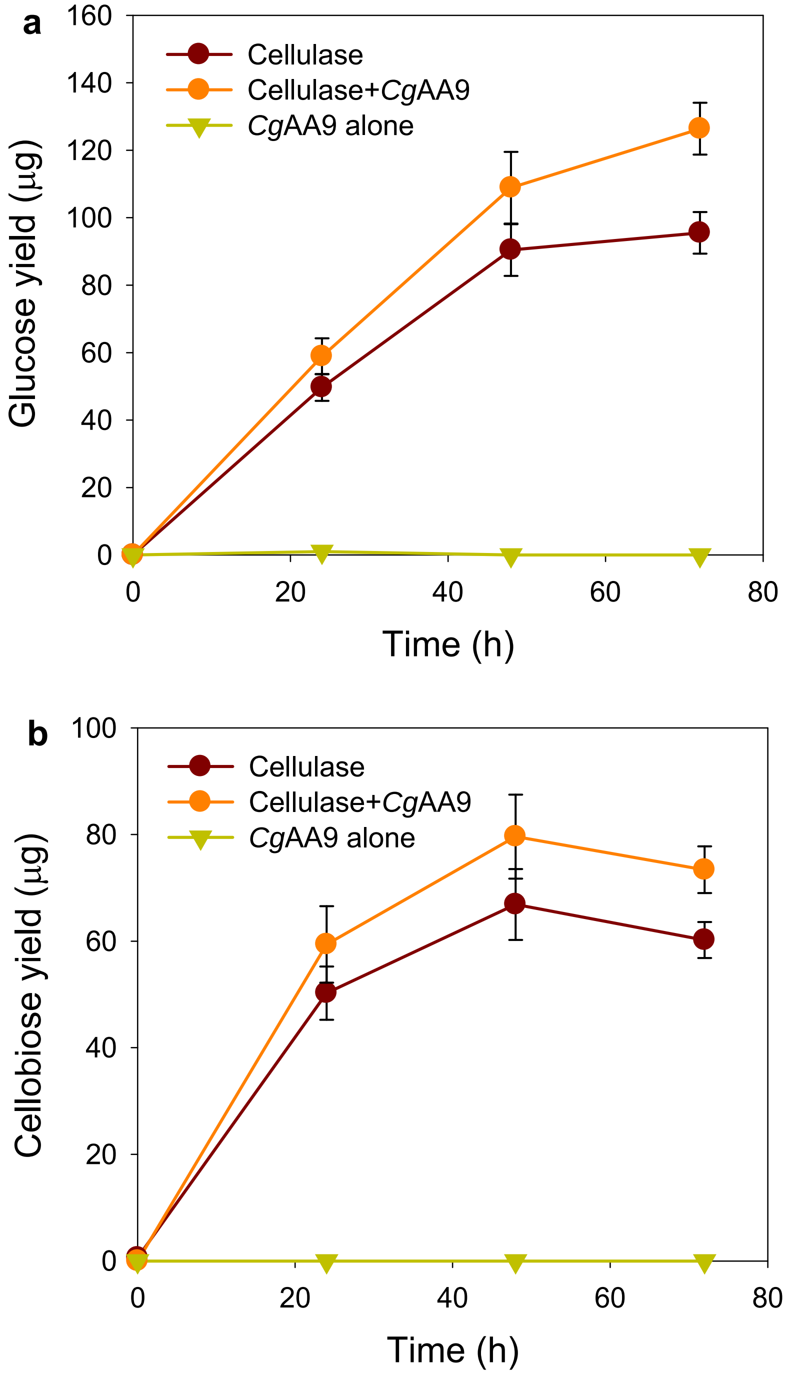

Fig. S3 Synergism of *Cg*AA9 in the cellulose hydrolysis by cellulase. The amounts of **a** glucose and **b** cellobiose were quantified using HPLC. Avicel (1%, w/v) was hydrolyzed by 1.2 FPU of Celluclast 1.5 L/g Avicel with or without 1.8 mg of *Cg*AA9/g Avicel for 0, 24, 48, and 72 h at 50°C in 50 mM sodium acetate buffer (pH 5.0). Data are means ± standard errors
